# Sustained Decline in Hospitalisations for Anogenital Warts in Australia: Analysis of National Hospital Morbidity Data 2003–2020 †

**DOI:** 10.3390/tropicalmed9040079

**Published:** 2024-04-08

**Authors:** Harunor Rashid, Aditi Dey, Han Wang, Frank Beard

**Affiliations:** 1 Faculty of Medicine and Health, The University of Sydney, Sydney, NSW 2006, Australia; aditi.dey@health.nsw.gov.au (A.D.); frank.beard@health.nsw.gov.au (F.B.); 2 Sydney Infectious Diseases Institute, The University of Sydney, Westmead, NSW 2145, Australia; 3 National Centre for Immunisation Research and Surveillance, The Children’s Hospital at Westmead, Westmead, NSW 2145, Australia; han.wang@health.nsw.gov.au

**Keywords:** anogenital wart, Australia, genital wart, hospitalisation, human papillomavirus, human papillomavirus vaccine

## Abstract

In Australia, school-based human papillomavirus (HPV) vaccination was introduced initially for girls in 2007, and then also for boys in 2013. While studies have shown declines in the incidence of anogenital warts, there is a paucity of recent data analysis assessing the impact of vaccination on more severe disease. The National Hospital Morbidity Database of the Australian Institute of Health and Welfare (AIHW) hospitalisation admission data that included ICD-10-AM code A63.0 (‘anogenital warts’) as the diagnoses, for the years 2003–2020, were analysed to estimate hospitalisation rates per 100,000 mid-year population. The annual average hospitalisation rates per 100,000 population for anogenital warts in both genders combined in the age groups 10–19 years, 20–29 years, and 30–39 years decreased, respectively, from 16.9, 49.6, and 23.6 in 2003–2007 (pre-vaccine period) to 2.6, 15.2, and 14.6 in 2008–2020 (post-vaccine period), equating to declines of 84.7%, 69.4%, and 38.2%, respectively. Following the introduction of the boy’s vaccination, hospitalisation rates decreased further in the respective age bands from 4.3, 22.8, and 18.4 in 2008–2013 (early post-vaccine period) to 1.1, 9.3, and 11.7 in 2014–2020 (late post-vaccine period), equating to respective declines of 73.4%, 59.3%, and 36.4%. This analysis confirms that there is a substantial incremental decline in anogenital warts hospitalisations among Australians aged 10–39 years.

## 1. Introduction

Human papillomavirus (HPV) is responsible for malignant and non-malignant lesions in both sexes, mostly affecting the anogenital region, but also other parts of the body [1]. Some HPV types (e.g., 16, 18, 31, 33, 35, 39, 45, 51, 52, 56, 58, 59, and 68) are causally associated with the development of cancers of the cervix, anal canal, vagina, vulva, penis, head and neck [2], while other types (e.g., 6, 11, 40, 42, 43, 44, 54, 61, 70, 72, 81, and 89) are predominantly associated with non-malignant lesions, including genital warts [3].

Australia has been a world leader in HPV vaccination and was the first country to implement a fully funded national HPV vaccination program for girls (aged 12–13 years) via schools in 2007 using three doses of the quadrivalent HPV (4vHPV) vaccine, which provides protection against four HPV types, two of which are oncogenic (16 and 18) and the other two (6 and 11) which are mainly associated with anogenital warts [4]. There was also a community-based catch-up program for women up to the age of 26 years that concluded in 2009 [5]. HPV vaccination uptake in Australia has been relatively high since program inception, with coverage of at least one dose of HPV vaccine by 15 years of age reaching 84% in females in 2010, and 87% in females and 85% in males in 2020 [6,7].

There is evidence of a marked impact of HPV vaccination on genital wart incidence from GP encounter data in Australia [8]. The management rates of genital warts in a nationally representative set of general practices that formed the Bettering Evaluation and Care of Health (BEACH) program decreased by 61% in vaccine-eligible females from the pre-vaccine period (2002–2006) to the post-vaccine period (2008–2012) (4.33 per 1000 encounters to 1.67 per 1000 encounters) [8]. A mobile telephone survey in 2011 among randomly selected Australian females aged 18–39 years also showed a 41% decrease in self-reported genital wart diagnoses in vaccine-eligible females and a 64% increase in vaccine-ineligible females compared with corresponding estimates from a similar survey in 2001. This survey also found that 63.3% of females reporting a genital warts diagnosis were treated by a GP, 15.2% in hospital and 12.7% at a sexual health clinic [9]. But only a limited number of studies looked at the evidence on severe morbidity such as hospitalisations from a nationally representative dataset.

A study that used comprehensive national hospital admission data from Australia for the years 1999 to 2011 found that hospitalisation rates for anogenital warts decreased significantly among females aged 12–17 years with an overall reduction of 89.9% (95% confidence interval [CI], 84.4–93.4%) from 2006–2007 to 2010–2011. There was also a more modest but significant decrease among males with an overall reduction of 38.3% (95%CI, 27.7–47.2%) among men aged 18–26 years [10]. In 2013, the HPV vaccination program was extended to boys aged 12–13 years, with catch-up for those aged 14–15 years old until the end of 2014 [11]. The 9-valent HPV (9vHPV) vaccine replaced the 4vHPV vaccine in the National Immunisation Program (NIP) of Australia from February 2018 [12], expanding protection to an additional five oncogenic HPV types (31, 33, 45, 52, and 58) and was similarly provided via school-based programs for girls and boys at ages 12–13 years in a two-dose schedule spaced at a minimum of 6 months. Since February 2023, 9vHPV is given as a single dose in the NIP. Catch-up is available under the NIP from primary care, with upper age limit extended from 19 to 25 years of age in February 2023.

With these major changes in the NIP, we aimed to estimate the hospitalisation rates for anogenital warts in Australia by three major vaccine introduction periods: pre-vaccine (2003–2007), early post-vaccine period (prior to the extension of the vaccination program to males) (2008–2013), and late post-vaccine period (2014–2020). The rates were analysed by gender, age, jurisdiction, and Aboriginal and Torres Strait Islander status.

## 2. Materials and Methods

We used hospital admission data from the National Hospital Morbidity Database of the Australian Institute of Health and Welfare (AIHW) for this analysis. Data included age, gender, and Aboriginal and Torres Strait Islander status of hospitalised patients and hospital diagnoses coded using the International Statistical Classification of Diseases, Tenth Revision, Australian Modification; (ICD-10-AM). 

The population estimates used as denominators for rate calculations were obtained from the Australian Bureau of Statistics (cat no. 3101.0, release date 19 March 2020). All AIHW hospitalisation data that included ICD-10-AM code A63.0 (‘anogenital warts’) as the principal or any of the additional diagnoses, for the years 2003–2020, were included. 

Hospitalisation rates were calculated per 100,000 population over relevant 12-month periods using the corresponding mid-year resident population estimate. Rates were stratified by sex and by age group (<10 years, 10–19 years, 20–29 years, 30–39 years, and ≥40 years). To make the data meaningful without having excessive decimal points, all rates were rounded after one decimal point, and two decimal points were used for rate ratios and the 95%CI.

Complete data for both numerators and denominators for all Australian states and territories, and for both Aboriginal and Torres Strait Islander and non-Indigenous populations, were available for the period 2003–2019; however, for the year 2020, data were available only for half the year and so data were annualised for that year. Average annual hospitalisation rates in the pre-vaccine period of 2003–2007 were compared with those in two post-vaccine periods: 2008–2013 and 2014–2020.

Ethics approval for this study was granted by the Sydney Children’s Hospitals Network Human Research Ethics Committee, protocol 2019/ETH12453.

## 3. Results

Between 1 January 2003 and 30 June 2020, a total of 45,470 hospitalisations that had a diagnosis of anogenital warts (ICD-10-AM code A 63.0) were identified across Australia. Of those hospitalisations, 25,100 (55.2%) were in females and 20,370 (44.8%) in males. There were 1123 (2.5%) hospitalisations in Aboriginal and Torres Strait Islander people: 773 (68.8%) in females and 350 (31.2%) in males.

**Figure 1:**
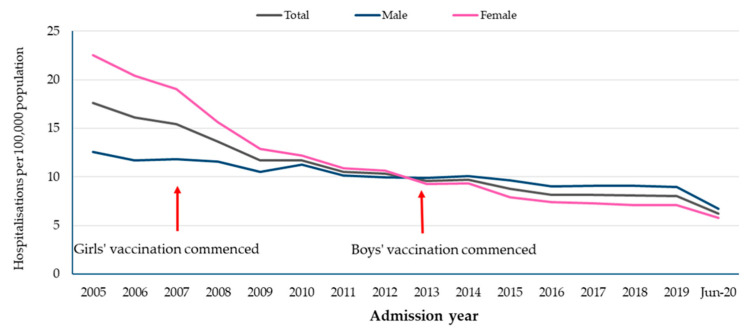
Anogenital warts hospitalisation rates (all ages) per 100,000 population by gender, 2003 to 2020.

Overall, there was a progressive decline in anogenital warts hospitalisations across the study period in both females and males. This decline was more pronounced in females (Figure 1).

### 3.1. Hospitalisations by Age and Gender

**Figure 2:**
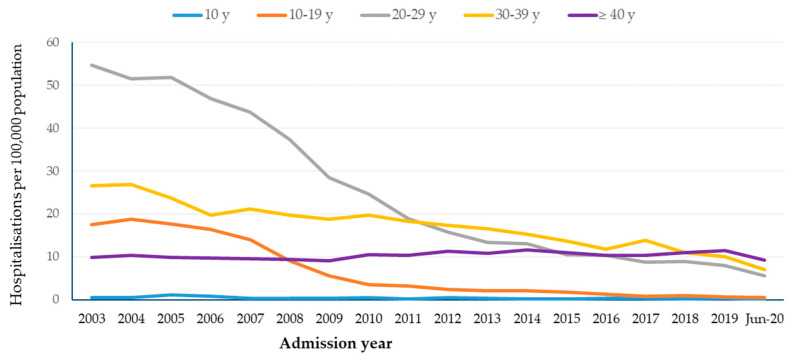
Anogenital warts hospitalisation rates per 100,000 population by age group, 2003 to 2020.

Overall, across both genders, there was a decline in hospitalisation rates in those aged 20–39 years (Table 1 and Table 2, Figure 2). Additionally, in females, there was reduction in hospitalisation in the age group 10–19 years. There was no significant change in rates in the <10 years and ≥40 years age groups. The greatest reduction in rate was in the 10–19 years age group followed by 20–29 and 30–39 years. Annual average hospitalisation rates per 100,000 population for anogenital warts in both genders combined in the age groups 10–19 years, 20–29 years, and 30–39 years in the pre-vaccine period (2003–2007) were 16.9, 49.6, and 23.6, and these rates decreased to 2.6, 15.2, and 14.6 in the full post-vaccine period (2008–2020), equating to declines of 84.7%, 69.4%, and 38.2%, respectively.

When compared with the early post-vaccine period (2008–2013), hospitalisation rates decreased in the late post-vaccine period (2014–2020), respectively, from 4.3, 22.8, and 18.4 to 1.1, 9.3, and 11.7 per 100,000 population in the age groups 10–19 years, 20–29 years, and 30–39 years, equating to declines of 73.4%, 59.3%, and 36.4%, respectively.

In females, the highest decline was in the 10–19 years age group, followed by 20–29 years. Annual average hospitalisation rates in the 10–19 years and 20–29 years age groups declined from 31.7 and 70.1 per 100,000 population in the pre-vaccine period (2003–2007) to 6.3 and 26.3 in the early post-vaccine period (2008–2013), equating to reductions of 80.3% and 62.5%, respectively. Hospitalisation rates in the late post-vaccine period (2014–2020) declined further to 1.2 and 6.7 per 100,000 population in the 10–19 and 20–29 years age groups, respectively, equating to a further 80.9% and 74.7% decline compared to the early post-vaccine period (2008–2013).

In males, annual hospitalisation rates in the years 2003–2013 (i.e., prior to the extension of the program to males) were 2.6 and 23.7 per 100,000 population in the 10–19 years and 20–29 years age groups, respectively; these rates decreased by 58.0% and 50.1% to 1.1 and 11.8 per 100,000 population in the 10–19 and 20–29 years age groups, respectively, in the years 2014–2020 (i.e., during the late post-vaccine period).

### 3.2. Hospitalisations by Indigenous Status

Among Aboriginal and Torres Strait Islander people, there was a significant decline in hospitalisation rates for anogenital warts from pre-vaccine (2003–2007) to post-vaccine periods (2008–2020) in the 10–19 and 20–29 years age groups (Table 1 and Table 3). However, a significant reduction was only seen among women overall and in the age group 10–29 years, and there was no significant change among Aboriginal and Torres Strait Islander men (Table 2). 

Among Aboriginal and Torres Strait Islander males aged 10–19 years and 20–29 years, hospitalisation rates in 2003–2013 (i.e., prior to the extension of the program to males) were 1.8 and 9.9 per 100,000 population, and these rates decreased by 64.5% and 15.8%, respectively, to 0.7 and 8.4 per 100,000 in 2014–2020 (i.e., during the late post-vaccine period).

### 3.3. Hospitalisation Rates by Jurisdiction

Hospitalisation rates for anogenital warts decreased over the years, from 2003 to mid-2020, in all Australian states and territories; however, the decline was more pronounced in some jurisdictions and age groups (Table 4). There were statistically significant reductions in the 20–29 years age groups in all jurisdictions except Northern Territory (NT), and in the 10–19 years age group in all except the Australian Capital Territory and NT, where the hospitalisation numbers were very small (Table 4). In states with larger populations (i.e., New South Wales, Victoria, Queensland, and Western Australia), there were also significant reductions in hospitalisations in the 30–39 years age group. In the <10 and ≥40 years age groups, there were no significant changes except in Queensland where there was actually a slight increase in rate in the ≥40 years age group in the post-vaccine period (Table 4).

## 4. Discussion

This analysis corroborates and confirms findings from previous studies that have showed a remarkable decline in hospitalisations for anogenital warts in Australia, and in incidence and non-hospital presentations, in the early post-vaccine period [8,10,13,14,15,16,17,18]. Our data also demonstrate ongoing substantial incremental declines in anogenital warts hospitalisation following the extension of the HPV vaccination program to males and gradually rising female coverage [7,19]. The decline observed in this study among females in age groups 10–19 and 20–29 years appears comparable to the findings of Smith and colleagues’ study that compared admission rates for anogenital warts for the pre-vaccination period (July 2006 to June 2007) and early post-vaccination period (July 2010-June 2011) finding, respectively, >85% and >60% reductions in anogenital hospitalisations among females, respectively, aged 10–19 years and 20–29 [16]. Smith and coworkers, however, reported no significant reduction in hospitalisation rates in the age group 30–39 years at that early stage of the vaccination program, but we found a significant decrease of 38.2% in this age group in both sexes combined in our extended post-vaccine study period, indicating the sustained long-term effect of the vaccination program. 

To our knowledge, no other Australian study has reported anogenital hospitalisation rates as late as until the year 2020, but a study that compared new genital warts diagnoses among patients who attended a sexual health clinic in Australia before vaccination (2004–2007) and after vaccination (2007–2018) showed that across both genders, aged <21 years, 21–30 years, and 31–37 years, there was a significant reduction, and there was also a reduction in males aged >37 years, but not in females aged >37 years [20].

Our data are also in concordance with data from Europe and North America where a significant reduction in hospitalisations for genital warts has been noted in age groups eligible for vaccination [21,22,23,24,25]. These declines are believed to be a reliable marker of disease reduction due to HPV vaccination and associated herd protection [26,27].

In this analysis, for the first time in Australia, we found a significant decrease in hospitalisations for genital warts in the 30–39 years age group, likely because the earliest vaccinated cohorts have now reached that age. However, among Aboriginal and Torres Strait Islander people aged 30–39 years, the rate of hospitalisations in the post-vaccine period (2008–2020) remained unchanged when compared with the pre-vaccine period (RR 1.11; 95%CI: 0.53–2.35); this may be due to lower uptake of vaccination in Aboriginal and Torres Strait Islander people during the adult catch-up program in 2007–2009 than in non-Indigenous people [28,29]. Sexual health clinic-based studies showed there was no significant change in anogenital diagnoses either for females or males aged 31–69 years [20,30], so now that we have found a significant reduction in hospitalisation rate in the 30–39 years age band, at least among non-Indigenous Australians; this indicates the further success of the program. Interestingly, we note a significantly higher rate of anogenital hospitalisations among non-Indigenous people aged 20–29 years before the vaccine introduction compared to their Indigenous counterparts (50.5 vs. 24.8 per 100,000 population, RR 0.49 [95%CI: 0.32–0.73]). This difference became non-significant in the 2008–2013 period (23.07 vs. 14.52 per 100,000, RR [95%CI: 0.63, [95%CI:0.38–1.01]), and the difference almost disappeared in 2014–2020 (9.3 vs. 8.7 per 100,000 population, RR 0.93 [95%CI: 0.52–1.64]), further illustrating the positive effect of the vaccination program in the age band that had the highest hospitalisation rate in the pre-vaccine era [31].

In this study, compared to males, females, both non-Indigenous and Indigenous, had a more prominent decline in anogenital wart hospitalisations and across wider age bands. This is consistent with the earlier implementation of an HPV vaccination program for females, and results from earlier studies that used national hospitalisation data [10], private in-patient treatment data [14], and national sentinel data from sexual health clinics [15]. There was a similar and significant decline in hospitalisations between non-Indigenous and Indigenous females, however, and although a clear decline was noted among non-Indigenous males in the age bands 20–29 and 30–39 years, the decreases among Aboriginal and Torres Strait Islander men in these age groups were not significant. A previous study noted that the number of anogenital warts hospitalisations among Indigenous men was too small even prior to the vaccination program to perform any meaningful comparative analysis [10]. We did not find any significant change in hospitalisation rates in those aged ≥40 years, nationally and across the individual states except for Queensland, consistent with other studies that noted no significant decreases in older age groups [14,32].

Our analysis used large national data up to seven years after the extension of the HPV vaccination program to males, but it has some limitations. We did not explore program impact by either socioeconomic or geographic factors beyond state of residence, as we did not have access to postcode-level data. We were unable to assess the impact on men who have sex with men (MSM), as it was not possible to identify MSM through hospitalisation data, and it is known that MSM are 2.6 times more likely to have anal warts than penile warts [33]. Another limitation is that most anogenital warts do not result in hospitalisation and hence these data represent the most severe cases. Also, some individuals could have multiple episodes of treatment which cannot be identified as belonging to a single individual in these data. Finally, another important limitation is health care access issues, which could be particularly relevant to the Indigenous population given greater access barriers including remoteness, cultural stigma, and cost.

## 5. Conclusions

In conclusion, this analysis using a large national dataset shows a substantial incremental decline in anogenital warts hospitalisation rates in both males and females aged 10–39 years following the expansion of the HPV vaccination program to include boys. As of yet, no effect is seen in the age group ≥40 years. The effect of the transition to a single dose of the HPV vaccine for adolescents and young adults, introduced in early 2023 in Australia, should be monitored in the near future.

## Figures and Tables

**Table 1:** Anogenital warts hospitalisation rates per 100,000 population pre-vaccine (2003–2007) to post-vaccine (2008–2020) introduction, by Indigenous status and age group.

Age Group	2003–2007 (a)	2008–2020 (b)	Rate Ratio (b/a)	95%CI
All Australians				
<10 years	0.6	0.3	0.46	0.22–1.11
10–19 years	16.9	2.6	0.15	0.12–0.19
20–29 years	49.6	15.2	0.31	0.28–0.34
30–39 years	23.6	14.6	0.62	0.55–0.69
≥40 years	9.9	10.5	1.07	0.98–1.17
Total	17.1	9.5	0.55	0.53–0.58
Aboriginal and Torres Strait Islander				
<10 years	1.3	0.4	0.28	0.04–4.97
10–19 years	20.4	3.1	0.15	0.06–0.39
20–29 years	24.8	11.0	0.45	0.23–0.88
30–39 years	14.0	15.5	1.11	0.53–2.35
≥40 years	6.0	12.8	2.13	0.99–4.87
Total	11.8	7.8	0.66	0.47–0.93
Non-Indigenous				
<10 years	0.6	0.3	0.49	0.21–1.18
10–19 years	16.7	2.6	0.15	0.12–0.20
20–29 years	50.5	15.4	0.30	0.27–0.34
30–39 years	23.9	14.6	0.61	0.54–0.69
≥40 years	9.9	10.5	1.06	0.97–1.16
Total	17.3	9.5	0.55	0.52–0.58

a = hospitalisation rate in pre-vaccine period, b = hospitalisation rate in post-vaccine period, b/a = rate in post-vaccine period ÷ rate in pre-vaccine period.

**Table 2:** Anogenital warts hospitalisation rates per 100,000 population pre-vaccine (2003–2007) to post-vaccine (2008–2020) introduction, by gender and age group among Indigenous and non-Indigenous Australians.

Age Group	2003–2007 (a)	2008–2020 (b)	Rate Ratio (b/a)	95%CI
Aboriginal and Torres Strait Islander Male				
<10 years	0.7	0.3	0.46	0.01–7.39
10–19 years	2.1	1.1	0.54	0.05–13.07
20–29 years	10.7	8.8	0.82	0.27–2.85
30–39 years	12.7	11.1	0.87	0.24–2.59
≥40 years	3.9	10.5	2.72	0.65–8.62
Total	4.9	5.7	1.18	0.62–2.30
Aboriginal and Torres Strait Islander Female				
<10 years	2.0	0.4	0.22	0.01–3.75
10–19 years	39.5	5.1	0.13	0.04–0.36
20–29 years	38.8	13.4	0.34	0.15–0.77
30–39 years	15.2	19.8	1.30	0.47–3.42
≥40 years	8.0	14.8	1.86	0.68–4.49
Total	18.7	9.8	0.52	0.35–0.79
Non-Indigenous Male				
<10 years	0.4	0.2	0.53	0.12–2.18
10–19 years	2.8	1.8	0.62	0.38–1.04
20–29 years	30.3	15.4	0.51	0.44–0.59
30–39 years	21.4	13.9	0.65	0.55–0.77
≥40 years	10.6	11.5	1.09	0.96–1.23
Total	12.6	9.8	0.78	0.72–0.84
Non-Indigenous Female				
<10 years	0.8	0.4	0.47	0.16–1.44
10–19 years	31.3	3.4	0.11	0.08–0.15
20–29 years	71.2	15.3	0.21	0.19–0.25
30–39 years	26.3	15.2	0.58	0.49–0.68
≥40 years	9.3	9.5	1.03	0.90–1.17
Total	22.0	9.3	0.42	0.39–0.46

a = hospitalisation rate in pre-vaccine period, b = hospitalisation rate in post-vaccine period, b/a = rate in post-vaccine period ÷ rate in pre-vaccine period.

**Table 3:** Anogenital warts hospitalisation rates per 100,000 population in Aboriginal and Torres Strait Islander people and non-Indigenous populations, by age groups and pre- and post-vaccine periods.

Age Group	Aboriginal and Torres Strait Islander (a)	Non-Indigenous (b)	Rate Ratio (a/b)	95%CI
2003–2007				
<10 years	1.3	0.6	2.33	0.51–10.06
10–19 years	20.4	16.7	1.22	0.82–1.78
20–29 years	24.8	50.5	0.49	0.32–0.73
30–39 years	14.0	23.9	0.59	0.34–1.03
≥40 years	6.0	9.9	0.61	0.29–1.16
Total	11.8	17.3	0.68	0.54–0.86
2008–2013				
<10 years	0.6	0.4	1.53	0.19–11.57
10–19 years	4.7	4.3	1.08	0.49–2.27
20–29 years	14.5	23.1	0.63	0.38–1.01
30–39 years	14.7	18.5	0.79	0.45–1.36
≥40 years	11.7	10.3	1.14	0.75–1.79
Total	8.2	11.3	0.72	0.56–0.93
2014–2020				
<10 years	0.2	0.2	1.10	0.07–21.45
10–19 years	1.8	1.1	1.67	0.48–5.17
20–29 years	8.7	9.3	0.93	0.52–1.64
30–39 years	16.2	11.6	1.40	0.86–2.33
≥40 years	13.5	10.7	1.27	0.86–1.80
Total	7.5	8.2	0.92	0.71–1.18

a = hospitalisation rate in Aboriginal and Torres Strait Islander population, b *=* hospitalisation rate in non-Indigenous population, a/b *=* rate in Aboriginal and Torres Strait Islander population ÷ rate in non-Indigenous population.

**Table 4:** Anogenital warts hospitalisation rates per 100,000 pre-vaccine (2003–2007) and post-vaccine (2008–2020) introduction, by state/territory and age group.

Age group	2003–2007 (a)	2008–2020 (b)	RR (b/a)	95%CI
New South Wales				
<10 years	0.5	0.2	0.52	0.08–2.47
10–19 years	14.9	2.2	0.15	0.10–0.24
20–29 years	46.2	14.5	0.31	0.26–0.38
30–39 years	22.1	13.6	0.61	0.50–0.76
≥40 years	9.7	9.9	1.02	0.87–1.19
Total	16.0	8.9	0.56	0.50–0.61
Victoria				
<10 years	0.7	0.4	0.48	0.12–2.13
10–19 years	16.0	2.2	0.14	0.08–0.24
20–29 years	59.4	16.0	0.27	0.22–0.33
30–39 years	28.6	16.3	0.57	0.46–0.70
≥40 years	11.2	11.2	1.00	0.84–1.18
Total	19.9	10.2	0.51	0.46–0.57
Queensland				
<10 years	0.6	0.3	0.48	0.09–3.37
10–19 years	18.1	2.6	0.14	0.09–0.24
20–29 years	40.9	13.0	0.32	0.25–0.41
30–39 years	20.0	13.5	0.68	0.51–0.89
≥40 years	7.9	11.3	1.43	1.16–1.77
Total	14.7	9.3	0.63	0.56–0.71
Australian Capital Territory				
<10 years	0.0	0.3	0.82	0.02–41.10
10–19 years	11.1	0.2	0.02	0.01–1.63
20–29 years	28.5	8.0	0.28	0.10–0.72
30–39 years	15.5	7.2	0.46	0.17–1.58
≥40 years	6.6	6.3	0.95	0.37–2.26
Total	11.5	5.2	0.45	0.27–0.80
South Australia				
<10 years	0.3	0.2	0.59	0.01–7.59
10–19 years	17.0	3.1	0.18	0.07–0.41
20–29 years	52.0	13.8	0.27	0.18–0.40
30–39 years	21.8	14.4	0.66	0.41–1.02
≥40 years	9.4	8.6	0.91	0.66–1.27
Total	16.5	8.4	0.51	0.41–0.62
Tasmania				
<10 years	0.8	0.2	0.20	0.01–8.56
10–19 years	34.7	5.6	0.16	0.05–0.58
20–29 years	78.0	25.3	0.32	0.17–0.62
30–39 years	21.1	24.1	1.15	0.47–2.51
≥40 years	9.5	14.7	1.55	0.88–2.80
Total	21.6	14.3	0.66	0.47–0.93
Northern Territory				
<10 years	1.2	0.4	0.36	0.01–7.76
10–19 years	10.1	2.6	0.26	0.03–3.18
20–29 years	32.7	11.7	0.36	0.13–1.08
30–39 years	24.8	11.7	0.47	0.16–1.46
≥40 years	12.5	11.6	0.93	0.36–2.18
Total	15.7	8.7	0.56	0.33–0.98
Western Australia				
<10 years	1.0	0.3	0.29	0.03–2.61
10–19 years	21.7	4.0	0.18	0.10–0.33
20–29 years	53.2	20.0	0.38	0.28–0.50
30–39 years	25.7	15.7	0.61	0.43–0.86
≥40 years	11.8	11.1	0.94	0.72–1.22
Total	19.6	10.8	0.55	0.47–0.64

a = hospitalisation rate in pre-vaccine period, b = hospitalisation rate in post-vaccine period, b/a = rate in post-vaccine period ÷ rate in pre-vaccine period.

## Data Availability

The data are not publicly available due to ethical restrictions. Readers who wish to interrogate the raw data should apply to the Australian Institute of Health and Welfare (https://www.aihw.gov.au/) which owns and curates the data.

## References

[1] Brotherton J.M.L., Bloem P.N. (2018). Population-based HPV vaccination programmes are safe and effective: 2017 update and the impetus for achieving better global coverage. Best Pract. Res. Clin. Obstet. Gynaecol..

[2] Bouvard V., Baan R., Straif K., Grosse Y., Secretan B., El Ghissassi F., Benbrahim-Tallaa L., Guha N., Freeman C., Galichet L. (2009). A review of human carcinogens—Part B: Biological agents. Lancet Oncol..

[3] Garland S.M., Steben M., Sings H.L., James M., Lu S., Railkar R., Railkar R., Barr E., Haupt R.M., Joura E.A. (2009). Natural history of genital warts: Analysis of the placebo arm of 2 randomized phase III trials of a quadrivalent human papillomavirus (types 6, 11, 16, and 18) vaccine. J. Infect. Dis..

[4] Garland S.M., Brotherton J.M., Skinner S.R., Pitts M., Saville M., Mola G., Jones R.W. (2008). Human papillomavirus and cervical cancer in Australasia and Oceania: Risk-factors, epidemiology and prevention. Vaccine.

[5] Brotherton J., Gertig D., Chappell G., Rowlands L., Saville M. (2011). Catching up with the catch-up: HPV vaccination coverage data for Australian women aged 18–26 years from the National HPV Vaccination Program Register. Commun. Dis. Intell. Q. Rep..

[6] Hull B., Dey A., Menzies R., McIntyre P. (2013). Annual immunisation coverage report, 2010. Commun. Dis. Intell. Q. Rep..

[7] Hull B., Hendry A., Dey A., Brotherton J., Macartney K., Beard F. (2023). Annual immunisation coverage report 2021. Commun. Dis. Intell..

[8] Harrison C., Britt H., Garland S., Conway L., Stein A., Pirotta M., Fairley C. (2014). Decreased management of genital warts in young women in Australian general practice post introduction of national HPV vaccination program: Results from a nationally representative cross-sectional general practice study. PLoS ONE.

[9] Liu B., Donovan B., Brotherton J.M., Saville M., Kaldor J.M. (2014). Genital warts and chlamydia in Australian women: Comparison of national population-based surveys in 2001 and 2011. Sex. Transm. Infect..

[10] Smith M.A., Liu B., McIntyre P., Menzies R., Dey A., Canfell K. (2015). Fall in genital warts diagnoses in the general and indigenous Australian population following implementation of a national human papillomavirus vaccination program: Analysis of routinely collected national hospital data. J. Infect. Dis..

[11] Wilkinson E. (2012). Australia leads way on HPV vaccination in boys. Lancet Infect. Dis..

[12] Wnukowski-Mtonga P., Jayasinghe S., Chiu C., Macartney K., Brotherton J., Donovan B., Hall M., Smith D.W., Peterson K., Campbell-Lloyd S. (2020). Scientific evidence supporting recommendations on the use of the 9-valent HPV vaccine in a 2-dose vaccine schedule in Australia. Commun. Dis. Intell..

[13] Ali H., Donovan B., Wand H., Read T.R., Regan D.G., Grulich A.E., Fairley C.K., Guy R.J. (2013). Genital warts in young Australians five years into national human papillomavirus vaccination programme: National surveillance data. BMJ.

[14] Ali H., Guy R.J., Wand H., Read T.R., Regan D.G., Grulich A.E., Fairley C.K., Donovan B. (2013). Decline in in-patient treatments of genital warts among young Australians following the national HPV vaccination program. BMC Infect. Dis..

[15] Ali H., McManus H., O’Connor C.C., Callander D., Kong M., Graham S., Saulo D., Fairley C.K., Regan D.G., Grulich A. (2017). Human papillomavirus vaccination and genital warts in young Indigenous Australians: National sentinel surveillance data. Med. J. Aust..

[16] Smith M.A., Liu B., McIntyre P., Menzies R., Dey A., Canfell K. (2016). Trends in genital warts by socioeconomic status after the introduction of the national HPV vaccination program in Australia: Analysis of national hospital data. BMC Infect. Dis..

[17] Donovan B., Franklin N., Guy R., Grulich A.E., Regan D.G., Ali H., Wand H., Fairley C.K. (2011). Quadrivalent human papillomavirus vaccination and trends in genital warts in Australia: Analysis of national sentinel surveillance data. Lancet Infect. Dis..

[18] Fairley C.K., Hocking J.S., Gurrin L.C., Chen M.Y., Donovan B., Bradshaw C.S. (2009). Rapid decline in presentations of genital warts after the implementation of a national quadrivalent human papillomavirus vaccination programme for young women. Sex. Transm. Infect..

[19] Hull B.P., Dey A., Beard F.H., Menzies R.I., Brotherton J.M., McIntyre P.B. (2016). Immunisation coverage annual report, 2013. Commun. Dis. Intell. Q. Rep..

[20] Khawar L., McManus H., Vickers T., Chow E.P.F., Fairley C.K., Donovan B., Machalek D.A., Regan D.G., Grulich A.E., Guy R.J. (2021). Genital warts trends in Australian and overseas-born people in Australia: A cross-sectional trend analysis to measure progress towards control and elimination. Lancet Reg. Health West. Pac..

[21] Checchi M., Mesher D., Mohammed H., Soldan K. (2019). Declines in anogenital warts diagnoses since the change in 2012 to use the quadrivalent HPV vaccine in England: Data to end 2017. Sex. Transm. Infect..

[22] Cocchio S., Baldovin T., Bertoncello C., Buja A., Furlan P., Saia M., Baldo V. (2017). Decline in hospitalization for genital warts in the Veneto region after an HPV vaccination program: An observational study. BMC Infect. Dis..

[23] Di Martino G., Cedrone F., Di Giovanni P., Tognaccini L., Trebbi E., Romano F., Staniscia T. (2023). The Burden of HPV-Related Hospitalizations: Analysis of Hospital Discharge Records from the Years 2015-2021 from a Southern Italian Region. Pathogens.

[24] Flagg E.W., Torrone E.A. (2018). Declines in Anogenital Warts Among Age Groups Most Likely to Be Impacted by Human Papillomavirus Vaccination, United States, 2006–2014. Am. J. Public Health.

[25] Nygård S., Nygård M., Orumaa M., Hansen B.T. (2023). Quadrivalent HPV vaccine effectiveness against anogenital warts: A registry-based study of 2,2 million individuals. Vaccine.

[26] Drolet M., Bénard É., Pérez N., Brisson M. (2019). Population-level impact and herd effects following the introduction of human papillomavirus vaccination programmes: Updated systematic review and meta-analysis. Lancet.

[27] Steben M., Garland S.M. (2014). Genital warts. Best Pract. Res. Clin. Obstet. Gynaecol..

[28] Hull B., Hendry A., Dey A., Brotherton J., Macartney K., Beard F. (2019). Annual Immunisation Coverage Report 2017. Commun. Dis. Intell..

[29] Soares G.H., Sethi S., Hedges J., Jamieson L. (2022). Disparities in Human Papillomavirus vaccination coverage among adolescents in Australia: A geospatial analysis. Vaccine.

[30] Chow E.P.F., Carter A., Vickers T., Fairley C.K., McNulty A., Guy R.J., Regan D.G., Grulich A.E., Callander D., Khawar L. (2021). Effect on genital warts in Australian female and heterosexual male individuals after introduction of the national human papillomavirus gender-neutral vaccination programme: An analysis of national sentinel surveillance data from 2004–2018. Lancet Infect. Dis..

[31] Brotherton J.M., Heywood A., Heley S. (2009). The incidence of genital warts in Australian women prior to the national vaccination program. Sex Health.

[32] Read T.R., Hocking J.S., Chen M.Y., Donovan B., Bradshaw C.S., Fairley C.K. (2011). The near disappearance of genital warts in young women 4 years after commencing a national human papillomavirus (HPV) vaccination programme. Sex Transm. Infect..

[33] Chow E.P., Lin A.C., Read T.R., Bradshaw C.S., Chen M.Y., Fairley C.K. (2015). Ratio of anogenital warts between different anatomical sites in homosexual and heterosexual individuals in Australia, 2002–2013: Implications for susceptibility of different anatomical sites to genital warts. Epidemiol. Infect..

